# The Impact on Ambulance Mobilisations of an Increasing Age Profile of Telecare Service Users Receiving Advanced Proactive, Personalised Telecare in Spain—a Longitudinal Study 2014–2018

**DOI:** 10.1007/s41666-021-00108-5

**Published:** 2021-11-06

**Authors:** Wendy Hugoosgift Contreras, Ester Sarquella, Eva Binefa, Mar Entrambasaguas, Anette Stjerne, Peter Booth

**Affiliations:** 1 Tunstall Healthcare Group, Whitley, UK; 2 Tunstall Ibérica, Madrid, Spain; 3 Televida Servicio Sociosanitario, Barcelona, Spain; 4 Tunstall Denmark, Skanderborg, Denmark; 5 Ignetica Ltd, Northwich, UK

**Keywords:** Telecare, Telehealth, Proactive telecare, Home monitoring, Ambulance mobilisations, Person-centred

## Abstract

Advanced proactive personalised telecare services in Spain have helped service users to live independently in their own homes for longer. Concern was however noted regarding potential impacts on ambulance mobilisations as time in the service, and mean age at cessation, increased. The purpose of this study was to investigate these impacts.

A longitudinal study of a telecare service user population in Spain (*n* = 202.1 k to 247.9 k) was undertaken using anonymised operational data collected in the delivery of proactive and personalised telecare services over the period 2014–2018.

For the studied population, ambulance mobilisation on a per-person/per-annum (pp/pa) basis reduced despite the increasing age profile at cessation and with the characteristics of the population at registration remaining otherwise similar over the period. The study identified the positive correlation coefficient between ambulance mobilisations and service user’s dependency levels, and marginal negative correlation in older age bands.

In conclusion, the increasing age at cessation has not correlated with an increased proportion of higher dependency service users. Indeed, the share of those over 85 years in the high dependency level decreased. This indicates that the changes in the telecare service which appear to have contributed to increased time living independently may also have helped ensure those continuing to live independently remain in lower risk bands.

## Introduction

Spain is one of the leading countries [1–4] in the application of proactive and personalised telecare to help support frail and vulnerable service users to live safely and independently in their own homes for longer.^1^ In basic form such services provide a personal emergency response management capability reacting to user or sensor triggered alarms (referred to as reactive telecare). Commencing in the 1990s, Spain pioneered progressively more advanced telecare approaches to help proactively support service users’ physical and mental health conditions, as well as wider social situations such as isolation (referred to as proactive telecare). This has become more advanced over time, with more recent developments including risk assessment/stratification to enable support to be directed most efficiently to service user needs (referred to as personalised telecare). Further explanation of the different levels of telecare is included in Section 1.1 below.

Through the delivery of proactive and personalised telecare services, comprehensive operating and sensor-based data is necessarily collected. This represents a rich dataset to enable advanced and progressively predictive analytics to further support the development of the telecare service capability, as well as supporting allied research. Tunstall Televida^2^ is one of the largest providers of proactive telecare services in Spain and, as result, has one of the largest and comprehensive datasets for analysis.

Recent research1 based on the same data [1] and explained more fully in Section 1.2 has identified the increase in the average time that service users were able to live safely and independently with progressively more advanced support. As a result, despite service users having a very similar mean age and profile at the commencement of the telecare service (registration) linked to the statutory eligibility criteria, the mean age at cessation (most frequently due to either their death or transitioning to residential or other care^3^) increased steadily (Table 1).

**Table 1:** Mean age at registration and cessation 2014–2018 (*n* = 202 k in 2014 increasing to 248 k in 2018)

Mean age	2014	2015	2016	2017	2018	Mean	Range
At registration	79.75	79.92	79.95	79.86	79.89	79.88	0.20
At cessation	83.88	84.29	84.77	84.88	85.18	84.60	1.30

**Fig. 1:**
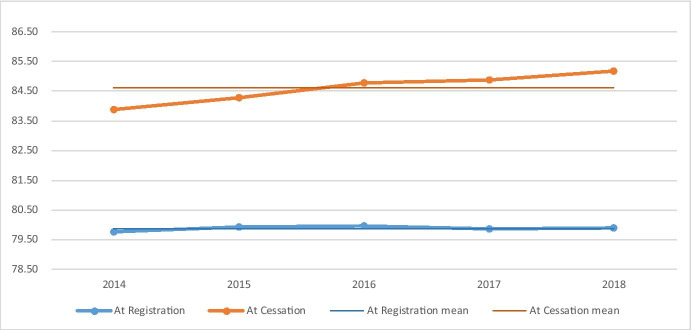
Mean age of service users at registration and cessation

As a result of the increasing mean age at cessation, concern was raised regarding the potential impact on ambulance mobilisations as an indicator of wider *emergency healthcare* demands (Fig. 1).

The purpose of this study was therefore to investigate the impact on ambulance mobilisations over the period *relative to the increasing mean age at cessation* including potential influencing factors including dependency, age band and gender shares in the service user population. To the best of our knowledge, this is the first study to be published which has specifically investigated the impact on ambulance mobilisations of these factors. The study does not seek to analyse the specific changes within the telecare service giving rise to the increasing length of stay in telecare and the consequent change in the mean age at cessation. The latter factors have been investigated in earlier research as described in Section 1.2 below.

### Different Levels of Telecare

**Fig. 2:**
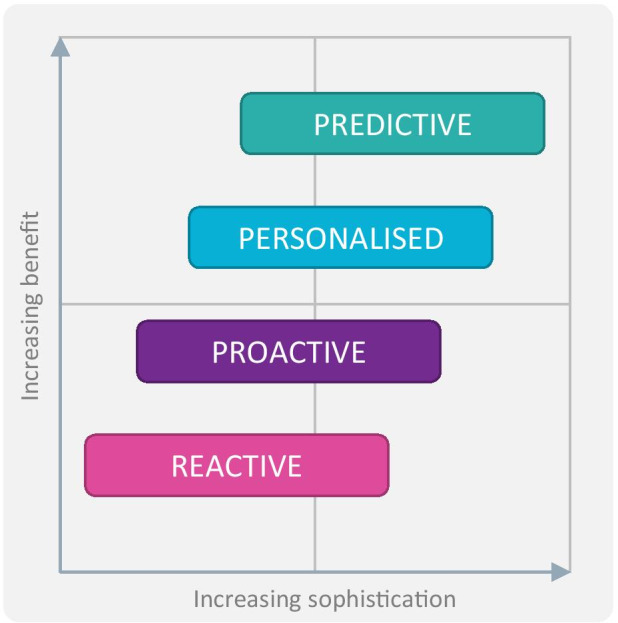
Levels of Telecare

In order to help define the different types of telecare services, a four-tier model was developed by Tunstall^1^, as set out below. Whilst this model is specific to Tunstall, it is consistent with wider themes of reactivity, proactivity and personalised telecare used within the industry globally, for example as used at The International Technology Enabled Care Conference, 2021^4^ (Fig. 2).

At the core of most telecare services is a **reactive capability** to respond to emergency situations. In the event of an issue being detected via installed sensors or via worn pendants, contact can be established with a monitoring centre which can then directly support, or mobilise the most appropriate help. Sensors may be tailored to the user’s specific situation to mitigate risks and combined with the reassurance of an effective response in the event of an issue, peace of mind can be provided for users and their friend/family carers.

**Proactive telecare** retains all of the aspects of reactive services but extends these in ways designed to avoid or reduce critical situations arising. Proactive telecare is normally delivered as an integrated programme of outbound calls, follow-ups, home care visits, along with advice and guidance, to provide broader and more holistic support for service users and their carers.

Proactive support can extend further when the service is **personalised** to the specific needs of the service user through an ongoing needs stratification process. As well as better meeting the specific needs of the users/carers, the approach enables support to be directed to those with the highest needs, risks and/or service usage.

The final tier in the hierarchy is advanced **predictive telecare** in which data-driven-insight and comprehensive health and care interventions can complement the proactive personalised telecare capability. By providing earlier predictive indications of developing issues, this offers the potential to inform earlier and more targeted interventions intended to help avoid the adverse event arising.

### Allied Research

This study was undertaken as part of the Tunstall research programme. Having identified the need for robust analysis of the benefits of different levels of telecare, the independent research programme, funded by Tunstall was developed spanning the reactive, proactive and personalised spectrum.

In the UK, the impacts on social care services of reactive telecare at Lancashire County Council (LCC) was analysed by the Yorkshire Health Economic Consortium (YHEC, part of the University of York) over the period 2016–2017. This identified the very significant costs avoided in wider social care costs through the application of telecare. Since reactive telecare is a core component of most telecare services, this insight is relevant not only in reactive telecare but also for all other telecare tiers.

In Spain, to investigate service user perceptions regarding user/family improvements in safety, self-sufficiency and peace of mind, a patient-reported outcome measures study [5] was undertaken in 2016 by the Foundation for Health and Ageing at the Universitat Autònoma de Barcelona (FSiE-UAB). This work identified the significant improvement in user-reported perceptions of their safety and self-sufficiency and increased peace of mind for their families.

Also in Spain, the benefit of proactive and personalised telecare was analysed at operational and economic levels by research consultancy Ignetica Ltd., based on Tunstall Televida service users between 2011 and 2018. In particular the work identified the increasing average time that service users were able to live independently with progressively more advanced telecare services. This study extends the research to now consider the implication on ambulance mobilisations of the increased time service users were able to live independently as reflected in the increasing mean age at cessation.

The key findings from each of these studies were published in “The transformational potential of telecare” Tunstall white paper in May 2020 [1].

### Trends in Ambulance Mobilisations

Demand for ambulance mobilisations is of interest in the first instance as a key emergency healthcare resource, but also as a key gateway to wider emergency healthcare. We are not aware of other studies which have investigated the specific impact on ambulance mobilisations of advanced telecare services which have enabled increased time living independently. However, existing literature has identified the increasing demand for ambulance mobilisations in general, and in older age groups specifically including comparisons between those living independently and in residential care facilities.

Ambulance utilisation and ambulance arrivals at emergency departments have increased in many developed countries over recent decades. As identified by Lowthian et al. [6], global examples include 7% annual growth between 2007/2008 and 2008/2009 for the London Ambulance Service, 25% growth between 1997 and 2005 in the USA and 20% growth between 2003/2004 and 2008/2009 in Canada.

More specifically, a further study considering emergency ambulance services in Melbourne Australia, Lowthian et al. [7] identified that the rates of mobilisations across all age groups increased from 32 per 1000 in 1994/1995 to 58 per 1000 in 2007/2008. With an annual growth rate at 4.8% (95% CI, 4.3–5.3%), this was beyond that explained by demographic changes alone [7]. The authors suggest alongside population ageing, changes in social support and accessibility/pricing as well as increasing community health awareness are all potential contributory factors. However, ageing is particularly significant with the same study identifying that patients over 85 years were 8 times as likely to be transported as those aged 45–69 years during the study period.

Adding to this insight, work by Dwyer et al. [8] considered patterns of emergency ambulance use for people over 65 years living in residential aged care facilities and the community (2009–2013) in Victoria, Australia. For 2013, this identified an average rate of ambulance use for those in residential care facilities at 788 (95% CI 784, 792) per 1000, approximately four times greater than the rate of 211 (95% CI 210, 212) per 1000 for those living in the community.

Whilst we were unable to identify existing literature analysing ambulance mobilisations for Spain specifically, these papers provide generalised background on rates and trends which may provide context for this analysis.

### Context for These Findings

The impact of *telehealth* technology on wider acute healthcare utilisation has of course been extensively analysed as highlighted in the recent Taylor et al. [9] systematic review evaluating the impact of remote patient monitoring on acute healthcare use. In other studies, the use of *telehealth* technology to enable initial remote assessments as part of emergency medical response prior to ambulance transportation has also been investigated [10, 11]. In contrast, this study investigated service users in receipt of progressively more advanced, social care-based, *telecare* services which did not include *telehealth* provision. As set out earlier, we are aware of no other studies which have investigated the impact on ambulance mobilisations of advanced proactive *telecare* and the increasing age of the services users supported to continue living independently. We believe this paper, therefore, contributes new insight which extends the existing research literature.

## Methods

This was a longitudinal study of the ambulance mobilisations of a telecare service user population over a 5-year period (2014–2018 inclusive) during which the mean age at cessation had increased by 1.3 years as set out in the introduction.

The population under study was the telecare service users of Televida Servicios Sociosanitarios, one of the largest providers of telecare services on behalf of regional municipalities in Spain. The population was subject to change over time as new service users became eligible to register for the service, and others ceased the service. Each of these factors was also studied to assess potential confounding or covariate factors in the population also influencing the mobilisation of ambulances.

### Data Sourcing and Processing

Anonymised data was provided by Tunstall Televida according to a data specification prepared as part of the study design to address the key demographic and operational metrics to be investigated. All data had been routinely collected in the course of the delivery of the telecare service.

The time series was selected on two basis: firstly, to consider the more recent changes in telecare proactivity including the development of a new registered^5^ personalisation/risk stratification assessment model in 2016 which may impact ambulance mobilisations and secondly, to avoid periods with confounding factors in the data. In 2013, there had been a high proportion of contract changes which influenced key variables, focussing from 2014 onwards avoided these factors.

In order to comply with the terms of use of the operational data, this was provided for analysis at mean annual levels for each of the variables requested. Although comprehensive this prevented analysis of distributions within the mean levels which may obscure important variations. In order to mitigate these issues so far as possible within this context, multiple segmentations spanning age band, gender and dependency level of the service users were also incorporated to assess for other potential variations in the data. The data specification was developed accordingly to define the data provision for each of these variables, segmentations and subsegmentations.

The data provided was comprehensive for all service users over the study period. As such no data was excluded in the analysis.

The risk stratification assessment model introduced in 2016 provided quantification of the dependency level of service users. For those service users who were in receipt of service prior to 2016, this was also retrospectively recorded for earlier years. However, for those who had left the service prior to this allocation then dependency data was unknown. In the analysis of the population in each dependency level, the ‘unknown’ proportion is highlighted, and the relative shares within those with known data also analysed. As described more fully later in this paper, some caution must be applied to the analysis of dependency for the period prior to 2016 based on these factors, and some analysis has therefore been narrowed to the 2016–2018 timeline accordingly. However, as eligibility for the service is subject to separate statutory dependency criteria, significant variation would not have been anticipated.

### Analysis of the Service User Population

Access to the telecare service is subject to regional government control and statutory eligibility criteria established by the ‘System for Promotion of Personal Autonomy and Assistance for Persons in a Situation of Dependency (SAAD)’ Act of 2006.^6^ The eligibility criteria did not change over the study period. However, in order to identify other potential influencing factors in new registrations, the overall service user population and those leaving the service (most frequently due to their death or transition to residential care) were analysed overall and by age band, gender and dependency level across the time series.

### Analysis of Ambulance Mobilisations

A core aspect of the telecare service^7^ is the management of personal emergency responses. In these events a contact centre triages the alarm, manages the case and mobilises the most appropriate response based on the situation. As a result, data is routinely collected on ambulance and other mobilisations for all service users.

This data has been analysed over the period in rates per person/per annum (PP/PA) broken down by age band, dependency level and gender. Where the analysis of the population indicated potential covariates, these were also investigated to assess their influence in the findings.

## Results

### Population Characteristics

As an active telecare service user population, this was subject to change over time as new service users became eligible to register for the services whilst for others the service was ceased, most frequently due to their death (43.8% of cessations over the 2014–2018 period), or transfer to residential care (25.9% of cessations over the period).

With new registrations in excess of cessations, the population grew year on year. With these changes taking place throughout the year, in the data an average number of service users over the 12 months was derived and reported (average active users). On this basis the total population in 2014 was 202,100 growing to 247,900 in 2018 as a result of 215,700 registrations and 161,900 cessations as summarised in Table 2. The mean annual population over the study period was 224,460.

**Table 2:** Population over the study period

Population (‘000)	2014	2015	2016	2017	2018
Average active users*	202.1	211.4	223.2	237.7	247.9
Cessations	30.1	31.5	31.4	33.6	35.3
Registrations	38.0	41.4	44.0	47.4	44.9
Growth rate (YoY)	3.3%	4.6%	5.6%	6.5%	4.3%
Cessation rate**	14.9%	14.9%	14.1%	14.1%	14.2%
Registration rate**	18.8%	19.6%	19.7%	20.0%	18.1%

*Average active users in the year reflects the timing of registrations/cessations and as such this is not simply additive of registrations less cessations. **Change in the year as a share of the average active users during the year.

The provision of telecare in Spain is subject to statutory and local governmental regulation. Until 2012 the programme was run at national level by the Institute for the Elderly and Social Services (IMSERSO) an agency of the Spanish Government, and subsequently under regional governmental control. Access is further controlled and entitlement governed by the System for Promotion of Personal Autonomy and Assistance for Persons in a Situation of Dependency (SAAD) Act 39/2006^8^ known as the Dependency Act. As such with no material changes in the eligibility criteria over the period the profile of service users registering for the service would be anticipated to remain consistent. However, to ensure that there were no other influencing factors in new registrations, these were analysed in terms of age band, gender and dependency level.

### Population Age at Registration

As illustrated in Table 1, and further expanded in Table 3, the mean age of service users at registration in each of the years has been stable with a mean of 79.88 years and a low to high range of 0.2 years (79.75, 79.95). For female service users for whom the mean age was 0.21 years younger at registration (79.67), the range was 0.2 years (79.57, 79.78) whilst for males the age was 0.49 years older (80.37) with a range of 0.45 years (80.09, 80.54).

**Table 3:** Mean age at registration overall and by gender (per annum and for the time series)

Mean age	2014	2015	2016	2017	2018	Mean 2014–2018	Range Years
At registration (all)	79.75	79.92	79.95	79.86	79.89	79.88	0.20
> Female	79.60	79.72	79.78	79.57	79.67	79.67	0.20
> Male	80.09	80.31	80.54	80.51	80.41	80.37	0.45

### Population Gender Profile

The population gender profile has been broadly consistent with a mean share of 73.3% female and 26.5% male over the period; however, there has been a small but progressive reduction in the female share year on year, reducing by 1% point in total from 2014 to 2018 (Table 4).

**Table 4:** Service user population shares per annum

Share of population	2014	2015	2016	2017	2018	Mean 2014–2018	Range % point
Female	73.9%	73.6%	73.4%	73.1%	72.9%	73.3%	1.0%
Male	26.0%	26.3%	26.5%	26.8%	27.0%	26.5%	0.9%

### Population Dependency

The dependency of the service users has been routinely assessed using a registered^5^ assessment and stratification methodology since it was introduced in 2016. This was designed by Tunstall Televida with the advice of the Fundación Salud y Envejecimiento of the Universidad Autónoma de Barcelona (FSiE-UAB)^9^ and is specific to the telecare service. The main variables used to determine the level of needs and support are social relationships, environment support and self-perception of health status. Through this process risks are assessed into one of four dependency bands (level 1 at the lowest, level 2, level 3 and high risk at the highest level). These are used operationally as part of the personalised proactive telecare service development and delivery. For the purposes of this study the data also provides a basis of assessing changes in the needs of the population over time through the share of the population in each level. However, some caution is required due to the introduction of the scheme in 2016 midway through the time series. Existing service users who were in place in 2016 were allocated a level which is retrospectively reported for the preceding years. Service users in 2014 through to 2016 whose service ended prior to the methodology being introduced would not have an allocated level, and as such in the earlier period there is a significant proportion of the overall population (27.7% in 2014) for whom the level is unknown. However, by assessing the shares of the population in each band relative to the population that does have a defined dependency level (i.e. excluding those with unknown or not applicable banding), the relative trends can be seen as per Table 5.

**Table 5:** Service user dependency levels as a share of the total population with defined levels

Dependency level	2014*	2015*	2016*	2017	2018	2014–2018 mean
Level 1	57.3%	58.0%	58.5%	59.3%	59.6%	58.7%
Level 2	37.5%	37.0%	36.5%	35.9%	35.6%	36.4%
Level 3	4.8%	4.7%	4.6%	4.3%	4.3%	4.5%
High Risk	0.4%	0.4%	0.4%	0.4%	0.4%	0.4%
Unknown (% Total)**	*27.7%*	*18.0%*	*8.1%*	*0.7%*	*0.1%*	*11.8%*
NA (% Total)**	3.7%	3.9%	4.0%	3.4%	2.7%	3.5%

*Shares reflect allocations made in 2016 onwards for service users in place prior to this point. **As a share of the total active service user population in the year excluding Unknown and NA. “Unknown” relates to those service users for whom dependency data was not recorded implying they are services users who had primarily left prior to allocation of ratings in 2016—it is for this reason that they fall rapidly from 2016 onwards. NA indicates instances where dependency data is defined but is recorded as being *not available*, these are most likely to relate to very short stay service users or those newly registered but not yet fully assessed.

The share of the population with known dependency levels is broadly consistent per level, particularly in the post 2016 period when allocations were current rather than retrospective.

Reflecting the basis of dependency data pre 2016, the unknown cohort, taken as a proportion of the entire service user population, is highest for 2014 and decreases year by year. This remained significant in 2016 as the new mechanism was introduced during the year, but in the subsequent years it reduced to 0.7% and 0.1% by 2018.

### Population Dependency by Age Band

Table 6 analyses the service user population by share in each age band overall and for each dependency level. This has been compiled for the shortened timeline 2016–2018 reflecting the years within the time series where the dependency levels were directly used and excluding the years 2014–2015 for which the allocation was retrospective.

**Table 6:** Share of service users in each dependency level broken down by age band mean 2016–2018

2016–2018 mean	Age bands						
	< 60 years	60–64 years	65–69 years	70–74 years	75–79 years	80–84 years	> 85 years
All levels	1.56%	0.86%	1.51%	4.01%	10.28%	23.64%	58.09%
Level 1	1.36%	0.70%	1.29%	4.02%	11.04%	25.32%	56.26%
Level 2	1.79%	1.04%	1.90%	4.90%	11.22%	23.95%	55.20%
Level 3	2.51%	2.00%	3.07%	7.39%	13.37%	23.92%	47.75%
High risk	5.66%	2.77%	5.25%	9.08%	17.77%	24.56%	34.90%

As can be seen, the population is heavily skewed towards the 80 and over age bands and this also heavily influences the consequent breakdown for each of the dependency levels. As noted in Table 5 the distribution of dependency levels is such that very small proportions of the population are in the higher bands (e.g. approximately 5% in level 3 and high risk combined) compared with approximately 95% in levels 1 and 2. The breakdowns of the shares of all services users within each band should therefore be interpreted within the relative size of that band as presented in Table 5.

### Mean Age at Cessation

As indicated in the earlier research [1], the mean age at cessation of the telecare service increased across the time series (Table 7) increasing by 1.3 years overall. For female service users this was higher at 1.48 years and lower for male service users at 1.02 years.

**Table 7:** Mean age at cessation overall and by gender (per annum and for the time series)

Mean age	2014	2015	2016	2017	2018	2014—2018 mean	2014–2018 change
At cessations (all)	83.88	84.29	84.77	84.88	85.18	84.60	1.30
> Female	84.06	84.57	85.10	85.29	85.54	84.91	1.48
> Male	83.59	83.83	84.20	84.21	84.61	84.09	1.02

**Fig. 3:**
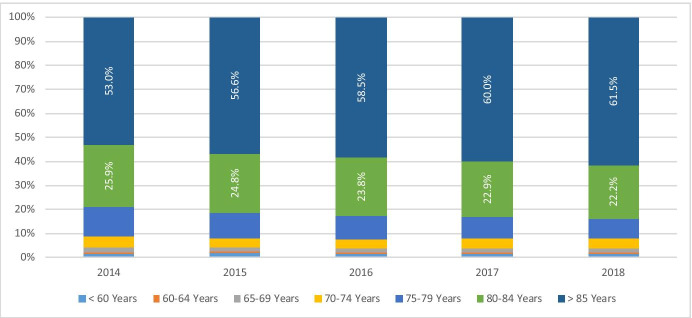
Share of cessations by age band

Analysis of the share of cessations by age band (Table 8) provides further information on the underlying change in the age profile. Year on year the proportion of service users who have ceased the service at over 85 years of age has increased from 53.0% in 2014 to 61.5% in 2018 (an increase of 14.5% points). Correspondingly, the shares in other bands have reduced with the greatest reduction in the 80–84 band and smaller changes in the younger age bands through to 65–69. For those 60–64 years there was an increase of 0.1% points whilst those in the less than 60-year bands reduced by 0.3% points (Fig. 3).

**Table 8:** Share of cessations by age band

Age band at cessation	2014	2015	2016	2017	2018	2014–2018 change
< 60 years	1.6%	1.7%	1.4%	1.5%	1.4%	− 0.3%
60–64 years	0.8%	0.8%	0.8%	0.9%	0.9%	0.1%
65–69 years	2.0%	1.9%	1.7%	1.7%	1.7%	− 0.3%
70–74 years	4.6%	3.8%	3.7%	4.1%	4.0%	− 1.6%
75–79 years	12.0%	10.4%	9.9%	8.9%	8.3%	− 5.6%
80–84 years	25.9%	24.8%	23.8%	22.9%	22.2%	− 6.7%
> 85 years	53.0%	56.6%	58.5%	60.0%	61.5%	14.5%

The factors contributing to the changes in age profile are addressed in the earlier research [1] and are not the subject of this study. However in outline the telecare service progressively developed in sophistication over the time series including greater use of sensor technology, more advanced proactive service availability and delivery of these on a personalised basis. The latter being influenced particularly by the introduction of the risk assessment stratification methodology^6^ in 2016. Although there are multiple factors involved in the increasing level of sophistication, the volume of outbound proactive telephone calls provides a partial analogous indication of proactivity and this was also studied as summarised in Table 9.

**Table 9:** Outbound calls per person per annum overall and per dependency band

Outbound calls pp/pa	2014	2015	2016	2017	2018	2014–2018 change
All service users	23.87	24.80	26.84	24.82	23.04	− 0.83
Level 1	20.59	21.83	24.18	21.78	19.18	− 1.41
Level 2	22.50	23.69	26.73	27.20	26.17	3.67
Level 3	27.78	29.52	34.46	44.86	57.93	30.15
High risk	28.43	28.82	37.05	50.78	67.42	38.99

The overall number pp/pa increased between 2014 (23.86) and 2016 (26.84) before reducing in subsequent years following the introduction of the risk stratification and personalisation methodology. The latter enabled activity to be focussed more directly on those with the highest needs, which is reflected in the very significant increase for high risk and level 3 bands, and more modest increase at level 2. Conversely level 1 reduced as those with the lowest needs did not require the same level of support. Given the higher proportion of service users in level 1, the overall number of calls pp/pa also reduced by 0.83 over the period.

### Ambulance Mobilisations

#### All Mobilisations

**Fig. 4:**
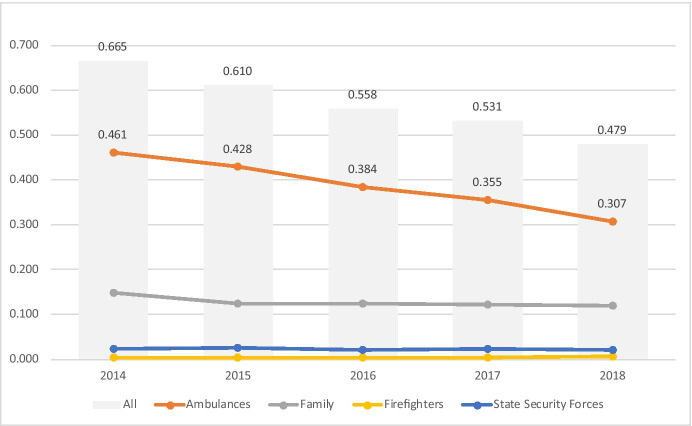
Mobilisations per person per annum (pp/pa)

Ambulance mobilisation was analysed on a pp/pa basis for each of the years along with all other mobilisation types, as shown in Table 10. Overall mobilisations reduced by 27.9% (0.665 to 0.479 pp/pa) over the period whilst for ambulances there was a reduction of 33.3% (0.461 to 0.307 pp/pa). There were also smaller reductions in the number of family and state security mobilisations. Firefighter responses increased by a large percentage; however, the absolute numbers pp/pa were by far the smallest at 0.003 mobilisations pp/pa in 2014 rising to 0.006 in 2018 (1.25% of mobilisations in that year) (Fig. 4).

**Table 10:** All mobilisation types, number per person per annum

Per person/per annum	2014	2015	2016	2017	2018	Change
All mobilisations	0.665	0.610	0.558	0.531	0.479	− 27.9%
Ambulances	0.461	0.428	0.384	0.355	0.307	− 33.3%
Family	0.147	0.125	0.123	0.121	0.118	− 19.9%
Firefighters	0.003	0.003	0.003	0.004	0.006	96.2%
State security forces	0.023	0.025	0.020	0.022	0.020	− 11.2%

#### Ambulance Mobilisations by Age Band

**Fig. 5:**
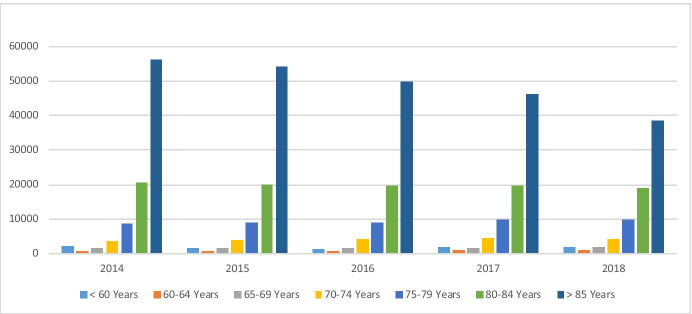
Total ambulance mobilisations by age band

**Fig. 6:**
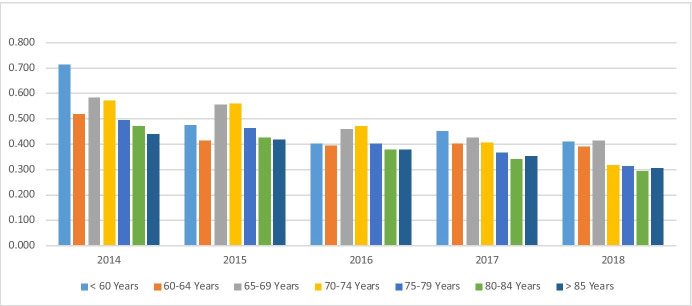
Ambulance mobilisations per person/per annum, by age band

Analysed by the numbers pp/pa per age band (Table 11) several observations can be made. In addition to the overall 33.3% reduction in ambulance mobilisations pp/pa, there are also reductions in each of the age bands. For the over 85 year band (− 31.0%) this is less than the overall rate (− 33.3%) but with 51.2% of the service user population in this band in 2018, the impact overall is the most significant. The reduction is least significant in the 60–64 years age band. It is also notable that over the period the numbers of ambulance mobilisations pp/pa do not generally increase significantly with age, particularly for those over 70 years. Conversely the largest number of ambulance mobilisations is frequently highest in the under 60-year band and more consistently in those under 70 years. However these represent much smaller proportions of the population and as such the absolute number (rather than pp/pa) of mobilisations per annum from this cohort is less significant, whereas for those over 85 s (as the largest cohort at 51.2%) are the most sizeable (Figs. 5 and 6).

**Table 11:** Ambulance mobilisations pp/pa by age band and relative to the population shares in 2018

	2014	2015	2016	2017	2018	2014–2018 change	% Pop 2018*
All age bands	0.461	0.429	0.384	0.354	0.307	− 33.3%	100%
< 60 years	0.714	0.475	0.400	0.450	0.407	− 43.0%	1.8%
60–64 years	0.517	0.411	0.395	0.400	0.391	− 24.4%	1.0%
65–69 years	0.582	0.554	0.458	0.426	0.412	− 29.1%	1.9%
70–74 years	0.568	0.558	0.471	0.403	0.316	− 44.4%	5.3%
75–79 years	0.493	0.464	0.401	0.366	0.311	− 37.0%	12.8%
80–84 years	0.470	0.424	0.377	0.338	0.294	− 37.4%	26.0%
> 85 years	0.440	0.415	0.377	0.350	0.304	− 31.0%	51.2%

*Share of the average active population in 2018 in each of the age bands.

#### Ambulance Mobilisations by Dependency Level

When analysed by dependency level there is a direct correlation between the increasing level and the numbers of ambulance mobilisations pp/pa as shown in Table 12. In 2018, for the high-risk band the mean number of mobilisations was 0.996 pp/pa, whereas at level 1 this was 0.226. In all bands other than high risk, there has been a net reduction in mobilisations across the series. For level 1 the change (− 23.3%) applies to 56.9% of the population (in 2018) and is therefore the most significant change. For the high-risk band there was a reducing rate from 2014 to 2017 but an increase in 2018 means over the period there was a net 1.9% increase. At 0.4% of the population this cohort is however the least populous risk band.

**Table 12:** Ambulance mobilisations pp/pa by dependency and relative to the population shares 2018

PP/PA	2014	2015	2016	2017	2018	2014–2018 change %	% Pop 2018*
Total	0.461	0.429	0.384	0.354	0.307	− 33.3%	100.0%
Level 1	0.294	0.280	0.255	0.259	0.226	− 23.3%	58.0%
Level 2	0.420	0.420	0.415	0.427	0.399	− 5.1%	34.6%
Level 3	0.818	0.720	0.691	0.745	0.746	− 8.8%	4.2%
High risk	0.948	0.931	0.822	0.796	0.966	1.9%	0.4%
Unknown	0.642	0.661	0.742	0.883	0.146	− 77.3%	0.1%
NA	0.812	0.829	0.734	0.565	0.116	− 85.7%	2.7%

*Share of the average active population in 2018 in each of the age bands.

The NA category has the highest percentage decrease at 85.7% with a relatively small share of the population (2.7%) by 2018. Those with *unknown level* have the second-highest reduction; however, as noted earlier this categorisation reduced from 27.7% in 2014 to 0.1% in 2018 and is related to the retrospective analysis of allocations made from 2016 onwards as noted earlier, rather than other operational factors.

#### Ambulance Mobilisations by Gender

Mobilisations of ambulances for female service user reduced by 35.8% (0.489 to 0.314) compared with 24.0% (0.384 to 0.292) for male services users. Each gender ambulance mobilisation reduced year on year and maintained a differential with higher rates of mobilisation for females. The scale of the differential reduced over the period from 0.384 cf 0.489 (males at 78.5% of female rate) in 2014, to 0.292 cf 0.314 (males at 95.1% of female rate).

As noted in Section 3.1.2 (population gender profile) there was a 1% point reduction in share of the female population from 73.9% (2014) to 72.9% (2018). This was analysed to assess the extent to which it may have contributed to the overall reduction in mobilisations.

A scenario was therefore modelled in which the female share of the overall population was maintained at 73.9% as per 2014, and the pp/pa mobilisations per gender applied to the modelled numbers per gender. As set out in Table 13, this suggests that in 2014 and 2015 there would have been no impact, and in 2016, 2017 and 2018 it accounted for 0.001 ambulance mobilisations pp/pa relative to the total of 0.384, 0.354 and 0.307, respectively. As such it appears that the overall ambulance mobilisations are not significantly impacted by the change in population gender mix.

**Table 13:** Ambulance mobilisations pp/pa by gender and relative to the population shares 2018

pp/pa	2014	2015	2016	2017	2018	2014–2018 Change %	% Pop 2018*
All	0.461	0.429	0.384	0.354	0.307	− 33.3%	100%
Man	0.384	0.356	0.326	0.323	0.292	− 24.0%	27.0%
Woman	0.489	0.455	0.406	0.366	0.314	− 35.8%	72.9%

## Discussion

### Findings

The findings indicate that for service users in the observed population, with broadly consistent profile at registration but an increasing mean age at cessation, ambulance mobilisations pp/pa reduced over the period by 33.3% (Table 10).

Further analysis of mobilisations by age band (Table 11) shows reductions in the rates in each band over the period. However, it may be significant that particularly for those over 70 years of age there is a generally small negative correlation between age band and mobilisations; i.e. the older the age band the lower the rates of mobilisation pp/pa. There are some exceptions, for example in 2017 and 18 the rates for those aged 80–84 years (0.338 and 0.294, respectively) were less than for those over 85 years (0.350 and 0.304, respectively); however, the broad trend remained with those for 80–84 and over 85 being less than those in the 75–79 band (0.366 and 0.311).

In contrast, when mobilisations are analysed by dependency level (Table 12), there is more significant positive correlation coefficient between increasing needs and ambulance mobilisations. In 2018, these were 0.226, 0.399, 0.746 and 0.966, respectively, for levels 1, 2, 3 and high risk.

Further analysis of the population share by age bands highlights high proportion of the service user population in the 80 and over age bands which is reflected within each dependency level (Table 6). Accordingly this illustrates that the share of service users over 85 years is most significantly represented in the lowest dependency level 1 and this decreases as each dependency level increases. In all cases those over 85 years represent the largest age band share. However, when considered relative to the mean over the 2016–2018 time series^10^ then it can also be seen that the share of those over 85 years in the high dependency level 3 and high-risk level has reduced.

This suggests that as more people have been enabled to live independently for longer, there has not been a corresponding increase in risk profile. In the earlier study [1] management highlighted that the increasing age at cessation and allied delay in demand for residential care was not an objective but a natural consequence of more effective analysis of risk/needs and more comprehensive person-centred proactive support. Through this approach, those with higher needs might be expected to progress to residential or other care more rapidly, whilst those who can be supported for longer remain at lower dependency levels. Although this study does not investigate these potential causal factors, the pattern identified in Table 6 and the wider analysis appears consistent with this hypothesis. This may be an interesting area for further analysis in due course.

### Comparison with Mobilisation Rates in Other Studies

We are aware of no other studies which have investigated the impact on ambulance mobilisations of advanced telecare services which have contributed to service recipients being able to live independently for longer, with increased mean age at cessation as a result. However, other studies do provide indications of the rates and trends in ambulance mobilisations more generally as identified in 1.3.

Data was not available for ambulance mobilisation rates in the wider populations matching to the geographic areas of the service user population studied, and as noted in 1.3, literature review did not reveal studies more generally in Spain. However, the rates of ambulance mobilisations/responses seen in other countries provide indications of rates which provide some context. For example the rates identified by Dwyer et al. [8] for Victoria, Australia, in 2013, of 211 per 1000 for those over 65 living in the community, and 788 per 1000 for those is residential care appear logically consistent with those found in this study. With reference to Table 14, in 2014 (the nearest equivalent year) the mean rate across all service users in this study was 0.461 ambulance mobilisations per person, per annum (or 461 per 1000). Of course this cohort of service users, whilst living independently had sufficient care needs to warrant the provision of telecare services and as such it would be logical to assume that this may translate into increased demand compared with the general 65 years and over population. As such, this rate being above that identified in Australia for those 65 and over living in the community (0.211 or 211 per 1000) and below the equivalent in residential care (0.788 or 788 per 1000) is as might be logically anticipated suggesting a degree of commonality in gross demand.

**Table 14:** Modelled analysis of rates of mobilisation if the female share of the population from 2014 remained static over the time series (in the data it reduced by 1% point over the period)

	2014	2015	2016	2017	2018
Modelled	0.461	0.429	0.385	0.355	0.308
Actual	0.461	0.429	0.384	0.354	0.307
Variance	0.000	0.000	0.001	0.001	0.001

Dwyer et al. [8] further identified the rates of ambulance responses within a generally increasing demand trend for the 65 years and older age groups. This mirrored the increasing demand trend across the wider population noted by Lowthian in Australia [7] and more globally [6]. Relative to this, the reduction in rates found in this study from 0.461 in 2014 to 0.307 in 2018 (Table 12) is particularly notable. Clearly in order to evaluate this further, wider Spanish Ambulance data would be need to be sought which was not available for this study. However with the potential commonalities in mobilisation rates and broad generalities in demand patterns across the developed world [6], it may be a reasonable initial assumption that demand might be expected to have increased in Spain following similar patterns and driving factors.

### Strengths and Limitations

This has been a relatively large scale study (population 202,100 in 2014 and reaching 247,900 by 2018 as a result of 215,700 new registrations and 161,900 cessations—the mean annual population being 224,460) over a 5-year time series. There are however several notable limitations, strengths and areas for potential further study.

The operational dataset provides a robust basis for analysis with all data being routinely collected through the planning and delivery of services. This overcomes potential challenges in sourcing and linking other disparate social care and healthcare datasets to analyse these factors and provides rich data for analysis as well as operational service delivery. In order to comply with the terms of use and anonymization, the data extraction was provided for analysis at mean levels for each of the requested metrics and subsets (e.g. by age band, dependency, gender) for each year studied. Although comprehensive, this prevented distribution analysis of the changes within each year or other statistical tests. There is therefore a risk that the use of mean annual measures may obscure important variations within the data. The use of more extensive segmented analysis of mean annual rates per age band, gender and dependency level have been undertaken to mitigate these risks so far as possible, whilst maintaining compliance with the data provision stipulations. This revealed significant nuances within the segmented analysis but none which would highlight significant concerns regarding variations which may otherwise be hidden through the use of mean data. Nonetheless, this remains a notable limitation.

There are also limitations due to the dependency level data being retrospective for the period pre-2016. The analysis does not indicate significant variation in levels, and the majority of the population even at the start of the time series had a defined level (72.3%^11^); however, this is a limitation.

This study sought to investigate the impact on ambulance mobilisation of the increasing mean age of service users at the cessation of the telecare service. The changes in levels of proactivity, personalisation, the use of increasingly more sophisticated monitoring sensors, process optimization and management of the telecare services leading to the increased age at cessation are not the subject of this paper. These are addressed in part in the allied research [1]. It should however be noted that these involve complex change and for similar results to be achieved elsewhere each of these aspects would need to be addressed. We have not sought to extrapolate from the studied service user population to the wider population in part due to these factors. Nonetheless, the findings illustrate the situation as achieved for the studied population which may be relevant to others considering similar advanced telecare services. A logical future area of study would be a comparative analysis between the outcomes achieved in the studied population and other telecare populations in Spain.

The impact on ambulance mobilisations was studied both as a key emergency healthcare resource and as a gateway indicator to wider emergency healthcare demand. Future research to follow this pathway and consider the impact on emergency hospital admissions in terms of frequency and length of stay once admitted would be a natural progression. Similarly, further research is currently being undertaken by FSiE-UAB to assess the service users’ and carers’ perceptions via a patient-reported outcome measure study.

## Conclusions

This study illustrates that for the sample population of advanced telecare service users in Spain, the increasing mean age at cessation and higher proportions of the population over 85 years has not resulted in increased demand for ambulance mobilisations pp/pa. On the contrary, the mean level of mobilisations reduced over this time despite the otherwise similar population profile at registration for the service. The analysis further suggests that despite the increasing mean age, the proportion of those over 85 years with higher risk levels have been reduced with this contributing to the reduction in ambulance mobilisation levels.

## Data Availability

Data transparency is not applicable.

## Footnotes

1 IESE Study, PPP for Cities, Barcelona Telecare Programme (Spain) https://www.iese.edu/wp-content/uploads/2019/03/ST-0454-E.pdf, accessed 31/10/20.

2 Tunstall Televida (comprised of Tunstall Ibérica and Televida Servicio Sociosanitario) is the Spanish subsidiary of Tunstall Healthcare Group plc.

3 Over the 2014–2018 period the average share of cessation due to death of the service user was 43.8% a further 25.9% transitioning to residential care.

4 Telecare Services Association (TSA), International Technology Enabled Care Conference 2021, https://itecconf.org.uk/the-personalisation-of-health-and-care/

5 Registro Territorial de la Propiedad Intelectual, Consejería de Empleo, Turismo Y Cultura, Comunidad de Madrid, Doc 09/061396.2/15, NUMERO DE ASIENTIO REGISTRAL 16/2015/4532, Manual de uso de procedimiento modelo RET (respuesta eficiente en teleasistencia).

6 The System for Promotion of Personal Autonomy and Assistance for Persons in a Situation of Dependency (SAAD) Act 39/2006, Eligibility requirements available at https://www.imserso.es/sede_06/proc_sede/detalle_proc_sede/index.htm?id=12, also analysed at https://www.eurofound.europa.eu/data/tackling-undeclared-work-in-europe/database/law-to-support-care-of-dependent-people-spain

7 European Committee for Standardisation definition, CEN/TS 17,470:2020, ‘Social care alarm services’ available at https://standards.cen.eu/dyn/www/f?p=204:110:0::::FSP_PROJECT:67639&cs=100986BAF8F22DDF4996B89DAFBCB80D4, accessed 31/10/20.

8 The System for Promotion of Personal Autonomy and Assistance for Persons in a Situation of Dependency (SAAD) Act 39/2006, https://www.imserso.es/sede_06/proc_sede/detalle_proc_sede/index.htm?id=12, also analysed at https://www.eurofound.europa.eu/data/tackling-undeclared-work-in-europe/database/law-to-support-care-of-dependent-people-spain

9 FSiE-UAB, English Translation, Health and Ageing Foundation of the Autonomous University of Barcelona.

10 Chosen to exclude earlier periods when the level allocations were retrospective.

11 Data with unknown dependency levels was 27.7% of the population in 2014.

## Code Availability

Software application or custom code is not applicable.
